# Sick or Sad? A Qualitative Study on How Dutch GPs Deal With Sadness Complaints Among Young Adults

**DOI:** 10.3389/fsoc.2021.765814

**Published:** 2022-01-24

**Authors:** Eva L. van Dijk, Donald G. van Tol, Agnes D. Diemers, Albert W. Wienen, Laura Batstra

**Affiliations:** ^1^ Department of Child and Family Welfare, Faculty of Behavioural and Social Sciences, University of Groningen, Groningen, Netherlands; ^2^ Department of General Practice, University Medical Center Groningen, Groningen, Netherlands; ^3^ Department of Sociology, University of Groningen, Groningen, Netherlands; ^4^ Windesheim University of Applied Sciences, Zwolle, Netherlands

**Keywords:** general practitioner, depression, sick role, young adult, medicalisation

## Abstract

Feelings of sadness among young adults related to a certain phase of life or to societal factors run the risk of being interpreted as an individual medical problem. Therefore, healthcare professionals should more often widen their perspective and consider de-medicalization as being part of their professional responsibility too. This article presents results from a qualitative interview conducted with 13 GPs in different phases of their career to get more insight into the way they deal with complaints of sadness among young adults. All participants acted proactively but in different ways. Based on the interviews, a typology of three types of general practitioners has been created: the fast referrer, the expert, and the societal GP. There seems to be a paradox in the way GPs think about de-medicalization on a macro level and the way they act on a micro level. Elaborating on Parsons’(1951) classical concept of the sick role, this study introduces the term semi-legitimized sick role to clarify this paradox. The third type, “the societal GP”, appears to be the most able to show a more multifactorial view on complaints of sadness. Therefore, this type connects the most to a course of de-medicalization.

## Introduction

According to the World Health Organization, unipolar depressive disorders were ranked as the third leading cause of the global burden of disease in 2004 and is expected to move into first place by 2030 (Lépine and Briley, 2011).

The medicalization critique argues that the rise of depression globally exemplifies a process whereby a problem of living—indicating social origins and social contradictions—comes to be redefined as a problem of individual biology. The conceptual framework of medicalization has been mainly coined by social scientists, among them Peter Conrad (1992) who defined medicalization as: “Medicalization consists of defining a problem in medical terms, using medical language to describe a problem, adopting a medical framework to understand a problem, or using a medical intervention to ‘treat’ it” (p. 211). Critics like Illich (1975) who also take this view have argued that the biologization of depression constitutes a fundamental assault on the self, which, in the guise of a quick cure *via* the prescription of antidepressants, silences people’s dissent and diminishes their capacity to reflect upon the social and political roots of their affliction (Kitanaka, 2011). Another line of criticism asserts that the medicalization of depression has brought to North America a “loss of sadness” (Horwitz and Wakefield, 2007), whereby people are losing their capacity for tolerance, patience, suffering, and grief. Noting how emotional life is being transformed by the act of taking “happy pills,” some scholars suggest that this form of medicalization is creating moral anxiety—seen as impoverishing the cultural resources with which people have traditionally confronted the hardships of life (Chambers and Elliot, 2004). Parens (2013) also stressed that as medicine focuses on changing individuals’ bodies to reduce suffering, its increasing influence steals attention and resources away from changing the social structures and expectations that can produce such suffering in the first place. The abovementioned examples and effects of the concept of medicalization shares features with the concept of psychiatrization defined by Beeker et al. (2021) as a complex process of interaction between individuals, society, and psychiatry. In order to effectively criticize the medicalization and psychiatrization of a problem Kaczmarek (2019) indicates, one needs to find an alternative explanation and a solution that would be more adequate and helpful in a given situation.

Over the past decades, sociologists have shown that the medical profession is only one of the many engines driving the complex process of medicalization (Conrad, 2005). However, physicians do play an important role regarding this subject. After all, if someone is convinced that he or she is having a medical problem that a physician can solve, the physician has the authority to prove the opposite, consult other professionals or change the course (RvS, 2017). Next to individual factors, social factors may contribute to a higher risk of sadness complaints among young adults (RvS, 2017). The Dutch National Institute for Public Health and the Environment (Rijksinstituut voor Volksgezondheid en Milieu) (RIVM, 2018) emphasizes the pressure to perform that young adults could experience. There seems to be a tendency to want and meet high standards, for example, on social media (RvS, 2017). On the other hand, the fact that (young) people sometimes feel lost and insecure in their search for meaning, identity, and purpose in life is less accepted as “normal” nowadays (RvS 2017). In their reports, the Dutch Council for Health and Society (RvS, 2017) and The Health Council of the Netherlands (RIVM, 2018) warn against the overmedicalization in cases of complaints of sadness. They argue that healthcare professionals should more often widen their perspective and consider de-medicalization as being part of their professional responsibility too.

In the healthcare system in the Netherlands, the General Practitioner (GP) plays the role of the gatekeeper. That is why the GP is in most cases consulted first when people are dealing with complaints of sadness among other things. As primary care provides highly accessible services and secondary care is relatively expensive, recent changes in the Dutch healthcare system were aimed at a more eminent role for mental healthcare by general practitioners. Since January 2014, according to new referral criteria, patients with mild psychological symptoms or social problems should all be treated within general practices (Magnée et al., 2017). To accommodate GPs in their larger role in providing mental health care, from 2008, the Practice Nurse Mental Health (PN-MH) has been introduced in general practices. In order to encourage the shift from secondary to primary mental health care and to save costs, the Ministry of Health, Welfare and Sports decided to provide more financial means in 2012, 2013, and 2014 to stimulate the deployment of the PN-MH in general practices. The PN-MH provides support in general practice care to all patients with psychological, psychosocial, or psychosomatic symptoms, while working under the supervision of the GP (Trimbos-institute, 2014). The role of the PN-MH is rather new but a function and competence profile describes that the tasks of the PN-MH often include diagnostic clarification, screening, referring to other mental health caregivers and providing accessible mental health consultation and brief advice or short-term treatment based on motivational interviewing or psycho-education for patients with early signs of psychological disorders or social problems. As the PN-MH provides mental health consultation and brief advice or short-term treatment within general practice, the PN-MH can be easily reached and patients can be easily referred (i.e., low-threshold service) (Abidi et al., 2019). Research of Verhaak et al., 2013 shows that more patients with psychological problems or symptoms such as anxiety or depression are seeking treatment within general practice: in the first 6 months of 2014, there was a 21% increase in consultations for psychological diagnoses compared to the first 6 months of 2013. Furthermore, Magnée et al., 2017 show in their research that the introduction of the PN-MH has not decreased antidepressant prescriptions, but that it may have a postponing effect. Eventually, based on the results of the study of Abidi et al. (2019), it seems that the PN-MH does not contribute to increased chronic or acute alcohol abuse diagnoses. How the introduction of the PN-MH specifically can be placed in the broader light of medicalization and psychiatrization could be investigated in further other research.

In the Netherlands, 9% of the young adults were, in their own words, dealing with depression in 2017 (Cbs Statline, 2017). Considering the important role of the GP regarding these complaints and the risk of them being (over) medicalized**,** the aim of this study is to get more insight into the way general practitioners deal with this task in relation to the complaints of sadness among young adults, how those complaints are viewed by them, and how they are influenced by societal, personal, and professional factors and patient characteristics.

## Data and Methods

### Participants

To obtain more knowledge on how the change in the Dutch healthcare system in 2014 and years of work experience impact the GPs’ way of working when it comes to dealing with complaints of sadness, stratified sampling was used to select three specific groups of participants. A Dutch GP has received 3 years of specialist training after the basic 6 years of medical education. The first group were the alumni general practitioners, who finished their general practice after 2014 (abbreviated in Table 1 as A), the second group were the more advanced general practitioners (abbreviated in Table 1 as GP), and the third group were the general practitioners in training (abbreviated in Table 1 as GP-T).

**TABLE 1:** Participants characteristics.

(Group) nr	A1	A2	A3	A4	A5	GP1	GP2	GP3	GP4	GP5	GP-T1	GP-T2	GP-T3
Gender	Male	Female	Female	Female	Female	Male	Female	Male	Male	Female	Male	Male	Female
Work-experience as GP (in years)	2	2	3	2	1	20	16	18	26	6	—	—	—

To recruit the more advanced GPs, an invitation was delivered at their workplace, but because of the limited response of one reply, snowball purposive sampling was used to recruit the rest of the more advanced participants (GP). The other two groups, general practitioners in training (GP-T) and the alumni general practitioners (A), were recruited *via* the database Department of General practice of the University Medical Centre Groningen (UMCG). An invitation to participate was e-mailed to all alumni GPs who graduated in or after 2014 and to GPs who were in their third year of training. The e-mail included information about the research, pseudonymization of data, and information about whom to contact for questions about the study. One reminder was sent to them. Finally, 13 GPs participated in this study: 3 general practitioners in training, 5 alumni general practitioners who finished their general practice after 2014, and 5 more advanced general practitioners were interviewed. Professionals were informed again before the start of the interview about the general nature of the study and gave verbal consent. For characteristics of the participants, see Table 1.

In-depth semi-structured interviews were conducted with GPs (in training) of the Northern Region of the Netherlands between March and July 2017. Interviews were held in the participant’s workplace or home. Interviews lasted approximately 45 min.

**FIGURE 1:**
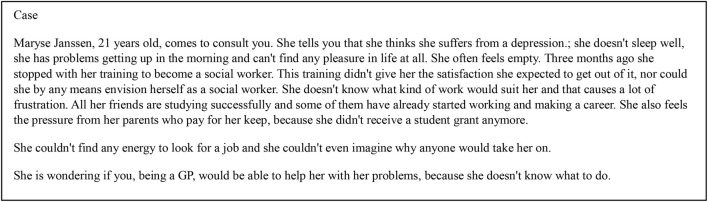
Vignette “Maryse”.

A vignette was used at the start of the interview to obtain information from the participants about knowledge, attitudes, and perceptions regarding the research topic. Subsequently, the societal, personal, and professional factors and patient characteristics were questioned, being the basis for the topics of the interview schedule. The vignette was developed by the researchers using their experiences and knowledge of psychiatry, general practice, and sociology. With utilization of the research tool of vignettes, information can be gathered on difficult or sensitive topics (Hughes and Huby, 2002). In this research, it represented a young adult with complaints of sadness (Figure 1). It is a fictitious scenario and is purely hypothetical. After the vignette was developed, several GPs revised the vignette to check the credibility.

All interviews were audio-taped and transcribed. For ethical reasons, pseudonyms were used in transcription to protect the identities of the interviewees quoted in this article. We stored the data securely so only the research team could gain access to it. A thematic analytical approach was applied, with the principles of grounded theory in mind (Strauss and Corbin, 1990) and with both inductive and deductive coding using Atlas.ti 8.4.3 by the first author. Analysis commenced with an “open” reading of the data to code the text. Axial coding through an iterative process was then conducted in accordance with the four constituent elements: societal, personal, and professional factors and patient characteristics. To enhance the validity of the analysis, a second author, AD, also coded the transcripts. The researchers compared their coding and discussed the differences until they reached consensus. The remaining interviews were coded according to the revised code list. Saturation was achieved after coding six interviews, since no new codes emerged. Furthermore, thick descriptions were made from the data by reading the data and delving deeper into each issue by exploring its context, its meaning, and the nuances that surround it. During the process, there were critical discussions in the research team to enhance the consistency and validity of the data.

## Findings

In what follows, we present our findings in three sections. First, we show the GPs’ responses on the presented vignette. Second, we demonstrate how their attitude towards complaints of sadness among young adults is influenced by societal, personal, and professional factors and patient characteristics. Third and summarizing, based on the findings, we show a drafted typology of GPs. We refer to the interviewed GPs using their group abbreviation and number.

### GPs Response on the Vignette

#### GPs’ Reaction

Tables 2–4 describe how the three groups of GPs (GP, A, and GP-T) respectively responded to the presented case in the vignette “Maryse” (Figure 1) and the corresponding questions: “what’s your first response,” “what’s your diagnosis,” “what kind of action would you undertake,” and other important notabilities. The participants responded very differently to the vignette. Some participants tended to diagnose the sadness complaints as depression, some were doubting but were almost sure of a depression, and others tended more to consider the complaints as life-phase problems. Remarkably, the participants’ first response to the vignette was milder. As the interviewed GPs read the vignette for the second time or when they paid more attention on how they would diagnose the complaints, their assessment became more serious. They tended to diagnose more depressive complaints or a depression:

“Yes, this is, this is a lady with feelings of insufficiencies, I think she’s not depressed. Maybe relatively sad. She thinks she has a depression…yes, ok… She is not sleeping well, it’s hard for her to get up in the morning. Ok. I think she is in it, ok. She feels empty. Yes there are symptoms of depression.” (GP4).

**TABLE 2:** GPs assessment and intervention.

	GP1	GP2	GP3	GP4	GP5
First response	Not pathological. A moment later: depressive symptoms. It does not “feel” like a depression	Does not want to label. Determines a few minutes later a “possible depression”	If the complaints exceed 2 weeks, she meets the diagnosis of depression according to the DSM	Thinks a depression is unlikely	Mood disorder
Diagnose	Using NHG standards. Not sure if symptoms meet the criteria “Maybe”	When the symptoms meet the NHG standards	A depression. Clearly, according to the DSM.	Does not use the NHG standards and DSM. No diagnosis by the GP.	Not yet.
Action	At first, normalizing. Follow up contact or consult at PN-MH.	First exclude physical causes. Consult PN-MH when symptoms meet NHG standards	Depending on the complaints PN-MH, a psychologist, or psychiatrist. Possibly start with antidepressants	Exclude physical causes. Further action depends on questionnaire completed by the patient	Follow up contact or consult at PN-MH for knowing the degree of severity
Other	Prescribes less antidepressants than 10 years ago. GP has also a societal function	Argues to take life phase problem out of the medical domain	Important to use DSM criteria for the common understanding; otherwise, there will be confusion	Sees GP more as a guide. Diagnose and prescribing antidepressants belongs to the task of a psychiatrist	Thinks there needs to happen more on societal level regarding the subject

**TABLE 3:** Alumni’s assessment and intervention.

	A1	A2	A3	A4	A5
First response	Being “stuck” because of more societal factors. Insomnia, eating problems, inactivity	Life phase problems. Logical questions on this age. Little later: depressive complaints	Complaints could fit a depression but they do not necessarily have to. Assessment is based on presentation, complaints and impression	Depression. When nothing happens, she will be in crisis in no time	“Quite” depressed not a “starting” depression
Diagnose	Depressive complaints. Could be or could become a depression. Uses NHG standards as a tool, not to diagnose	Suspicion of depressive complaints. Does not diagnose it herself. Instead, the PN-MH or the psychologist diagnose. Thinks the GP is only for an estimation	Needs to know more to diagnose	Depression according to the NHG standards and DSM. Already or very soon when nothing happens	Using the NHG standards for depression globally, only clearly for prescribing antidepressants
Action	Consult with PN-MH, job coach, social worker, or a psychologist when she wants	A questionnaire for the degree of severity of the complaints. Normalizing. Starting consultation at the PN-MH.	Assess the degree of severity him/herself or by the PN-MH. Psychologist is also an option	Start antidepressants. Refer to psychiatry, until that time consultation with the PN-MH to bridge the gap	Possibly an indication for the psychiatry
Other	Thinks that on a societal level there needs to be more attention for life phase problems	When it meets the DSM criteria for depression it is a depression. Also when there is a huge impact on life on the short term. Reluctant with antidepressants. Argues for more alternatives of the medical domain	Does not use the NHG standards to diagnose. Only sometimes to start with antidepressants	“Better to overstate than to understate.” This participant experienced a patient who committed suicide after she already referred this patient to psychiatry	Psychiatry is a sluggish system. More preferable is a consultation with the PN-MH or the psychologist. Prefers therapy over prescribing antidepressants

**TABLE 4:** GPs in training assessment and intervention.

	GP-T1	GP-T2	GP-T3
First response	Characteristics of depressive complaints, much less a depression, depending on the time and duration of it. “This demands action”	Meets the criteria of depression with underlying secureness. Could also possibly be a personality disorder	Life phase problems. Does not think the problem starts with a depression
Diagnose	Diagnose according to the NHG standards	Diagnose according to the NHG standards. Meets the suspicion of a DSM disorder	Assesses according to the DSM. Does not know if it means a depression according to the DSM, “could be”
Action	Consultation with the PN-MH or refer to a psychologist	Refer to a psychologist, in the meantime follow up at GP or PN-MH.	Coach or counsellor. Thinks this is a better way to deal with the problem
Other	Participant GP-T1 does not yet feel competent to deal with it him/herself. Reluctant with antidepressants	Reluctant with antidepressants	Ambivalent towards use of the DSM. At first, the participant concretely mentioned to use it; a moment later, this was contradicted

At first, eight participants—three more advanced GPs, three alumni, and two GPs in training—thought of related life phase problems or light depressed complaints when it comes to the presented case in the vignette. When the interviewer later asked them how they would diagnose the complaints of the patient in the case in the vignette, two alumni clearly answered that there are depressive complaints or a depression. One more advanced GP, one alumnus, and one GP in training answered that they did not know yet. Two more advanced GPs did not diagnose the case in the vignette. One of them also said that a GP is also not the right person to diagnose a depression.

Furthermore, seven participants—two more advanced GPs, three alumni, and two GPs in training—had the opinion that the girl in the vignette had a depression. When the interviewer asked when they diagnose someone with a depression, the participants answered that they rely on the criteria of the Dutch NHG standards^1^ or the Diagnostic and Statistical Manual of Mental Disorders (DSM).

Of the 13 participants, 11 mentioned the use of the DSM or the derived NHG standards to diagnose someone with a depression or to start antidepressants. The other two participants, two more advanced GPs, did not use the NHG but they did know the standard.

##### GPs’ Action

All participants said that they would undertake some form of action in response to the case in the vignette (Tables 2–4). None of the participants would send the patient from the vignette home empty handed. They all wanted to do at least something for the patient. As mentioned in the previous paragraph, six participants thought the patient from the case had a depression. One of them would immediately refer her to a psychiatrist. Another participant mentioned that it could be an indication for psychiatry. Four of the six participants who diagnosed the patient’s complaints as normal “life related” problems made a follow-up appointment or referred to their “physician assistant specialized in mental health.” Two participants suggested an approach outside of the medical perspective, for example, a coach or a counsellor. When it comes to prescribing antidepressants, it was remarkable that all the GPs in training and a few alumni were skeptical. They preferred a nonmedicinal therapy for sadness complaints among young adults.

### Factors Influencing GPs’ Attitude

Below, we will describe how GPs are influenced by societal, personal, and professional factors and patient characteristics in the way they are dealing with complaints of sadness among young adults.

#### Societal Factors

Most of the interviewed participants explained the increase of sadness complaints among young adults by pointing at societal factors*.* Societal “pressure” was mentioned frequently during the interviews. According to the interviewed GPs, societal pressure may lead to perceived high expectations among young adults, which expresses itself in a high pressure to perform. The high pressure to perform was also mentioned to be caused by government policies and the general multitude of choices nowadays.

“And also the binding recommendation on continuation of studies eh … and all those things. So they have to achieve a lot. And also … they have to look good, be slim, smart. So yeah well … A lot is asked of them, indirectly. So that has a little … well. If that doesn’t work out, you can feel like you have failed.” (GP3)

Also, technology and social media were mentioned 18 times by nine interviewed participants as a societal factor that may contribute to an increase of sadness complaints among young adults. According to the GPs, social media may lead to a disturbed perception of reality.

“And of course, well, it is because … Maybe social media plays a role in it too. Everybody has to share happy pictures. To prove that everything is fine with you. That completely drives you crazy, doesn’t it?” (GP4).

Furthermore, hedonistic characteristics and the way they were raised by their parents were mentioned as contributing factors to complaints of sadness among young adults.

#### Influence of Societal Factors on the Practice of the GP

A few of the interviewed participants spontaneously started to talk about medicalization. They talked about medicalization in a negative way and as something that needs to be prevented when it comes to sadness complaints among young adults.

“Yes, I think it’s very important to respond to phase of life issues and to de-medicalize it and … to try and keep the label of a depression diagnosis off. Because this label … will be remembered and will be taken along.” (A2)

The participants did not consider psychological treatment as a form of medicalization. They mentioned that a psychologist can help to deal with emotions, for example, through cognitive behavior therapy. Some interviewed participants mentioned that the medical domain is entered when visiting the GP, which can contribute to (over)medicalization. They argue that in order to avoid entering the medical domain with mild complaints of sadness, it is important to offer alternatives like community centers and schools. Other participants took opposite positions. When asked how they would de-medicalize, they seemed to struggle answering this question:

I: How do you try not to medicalize?

P: Ehm…. Yes, actually by naming it. By saying that I think that…. that the problem isn’t a disease or something like that. But more about how someone is dealing with, or isn’t capable to deal with more societal issues so to speak. But that’s difficult, because yes, not being able to deal with certain things (?) can also be… a problem you know. So yes, it’s complex, everything, has an impact on something else. You have to be aware of that in the sense that you look at the individual but you also take the background into account. And I think as a GP you need to do that, because you are the one who knows something about the background and you can ask things more easily.” (A3)

Reflecting on the identified struggle of GPs to deal with medicalization and to adopt a more de-medicalized attitude towards complaints of sadness among young adults, the GPs also mentioned the use of a nurse practitioner. Furthermore, they emphasized to focus on the patient’s own responsibility and to encourage patients to discuss problems with their family and friends.

When the interviewer introduced the statement of The Dutch Council for Health and Society (2017) on reducing the medical professional access in order to diminish medicalization and to encourage alternative professional perspectives, all participants agreed to this.

“In fact it would be a good thing when schools and universities would pay more attention to the subject. Ehmm … for example small scale education with coaching … more attention should be paid to this during studies in schools.” (A2)

During the interviews, all GPs reflected on their professional responsibility concerning sadness complaints. All GPs agreed that their responsibilities may transcend pure “medical” problems such as fractures or infections and explained that GPs are often consulted by patients for non-medical issues such as financial or relational problems or problems at work. Therefore, some of the interviewed GPs also see an important role for themselves on a more societal level. A few participants called it the “societal function” of the GP: “someone with whom you can discuss life questions and existential difficulties.”

“I am not only there for the medical function in a strictly medical domain. It’s more like … as you can see there are unfortunately less and less pastors, priests and that sort of people. We, the practitioners, adopted that function more or less naturally. People used to go to the pastor, the priest, depending on the religion one had; the imam … And with … with the secularisation of the world, the GP became one of the people who took that role.” (GP1)

#### Professional Factors

The interviewed participants frequently mentioned standards and guidelines when they explain how they are dealing with complaints of sadness among young adults. Of those, the NHG clinical practice standards^2^ are the most important in Dutch general practice. The NHG standard for depression is derived by the diagnostic criteria for depression in the Diagnostic and Statistical Manual of Mental Disorders (DSM IV). The NHG standards guide the GP in diagnosing and treating depression. The way the interviewed GPs used the NHG standards strongly differed. Most participants suggested that NHG standards are more or less “in their head,” meaning that they globally know the content of the NHG standards. They said not to use and follow the NHG standard exactly.

“Those are more books of reference. It’s not that we … it’s not a questionnaire. Well, of course in psychology and psychiatry there are questionnaires which are used to determine if someone has a depression or not and the seriousness of it … that is something we do not do in general practice.” (A3)

One participant did not use the NHG standards at all. Four other participants explained that they explicitly consult the NHG standards when prescribing medication. When asked about the difference between sadness complaints and a depression, four participants referred to the criteria for depression as stated in the DSM IV.

A second professional factor influencing the way the interviewed GPs deal with sadness complaints is experience. Three GPs stated that the more experience they had, the more they developed a more normalizing and de-medicalizing attitude towards complaints of sadness. Because of their experience, GPs feel capable to tell clients that their feelings are not weird or crazy and sometimes just part of life. Experience, usually connected with age, is a professional factor that results in a more normalizing attitude as well to a more moderate attitude towards medicalization:

P: Yes … I do think you de-medicalise more when you become older.

I: How come? Is it because you get older yourself or because you have become more experienced?

P: I think you recognise the relativity of the medical circuit, among other things. (GP1)

The more experienced participants called their own “gut feeling” important when they deal with complaints of sadness among young adults. Three GPs and one alumni talked among other things about an “intuitive” feeling. One GP, for example, remarked the following about the patient in the vignette:

Knowledge about complaints of sadness, which was closely connected to experience, also influenced the participants. In particular, the younger and more inexperienced participants (one alumnus and two GP residents) argued that they were not educated enough about complaints of sadness or light mental problems during their GP specialization/medical studies. This is why they did not feel fully competent to deal with this kind of problem.

Finally, from the interviews, it was also evident that there is a great “willingness to act” among the participants when it comes to the subject. Two participants explicitly argued that they would tell the patient in the vignette case that he or she wants to do “something,” to help and will do so. This also reflects the “struggle” the interviewed participants seem to have when it comes to complaints of sadness. Because of their “willingness to act” they tend to act proactively instead of adopting a more “wait and see” attitude.

“It’s obvious that we can do something, but first I would … euh, I would take stock of what the complaints are and euh(…) So … but I would certainly confirm I can do something. But in the first contact, that’s what I would do.” (A3)

#### Personal Factors

The personal curiosity and preference of the interviewed participants influenced the way they deal with young adults with complaints of sadness. Participants who had a lot of affinity with complaints of sadness showed a more active role than the participants who did not have much interest in the subject. Affinity could also impact the way one acts towards complaints of sadness:

“If I have known the patients for a long time, or if I’ve got a special interest in problem I tend to ‘keep them’ (i.e., patient to be treated by the GP). That’s better because they have also known me longer, then it’s easier for them. So yeah, then it’s a matter of how much affinity you have with the patient or with the problem. As a GP you always have to consider what you can do yourself or when to send to a specialist. If you had an endless amount of time you could do a lot more yourself. But you have to make choices. When you have affinity with them, you keep them with you longer. Yes, and of course the seriousness of the problem.” (GP1)

Affinity with the subject could cause a less fast referral to other disciplines, which is evident from the following quote:

“No … and I’m sure there are colleagues who quickly classify and then refer. But I just think it’s … euh … extremely interesting.” (GP5)

From the interviews, it turned out that participants used personal experiences to empathize with the patient, which could lead to a more empathic attitude. Normalizing complaints of sadness was also linked to one’s own life experiences. Some GPs explained that they were inclined to a “normalizing attitude” towards sadness complaints, when they recognized a patient’s problems from their own personal life. Younger participants, for example, recognized the issues young adults are dealing with from their own experiences as a student.

An interviewed GP, full of emotions, told that after she referred a young girl with complaints of sadness, the young girl committed suicide. This incident had a huge impact on the GP. Since that time, she referred almost every young adult with complaints of sadness to psychiatry or equivalents. The participant said:

“Yes maybe we refer more often and more fast. And … Uhh yes. Maybe I overestimate sometimes. But anyway, yes” (A4).

#### Patient characteristics

During the interviews, participants sometimes expressed particular ideas about young adults in general that seem to influence the way they deal with individual (young adult) patients. One participant, for example, said that it is popular among young adults to be sad and to “have” a psychologist. This led to a more skeptical attitude towards the problem.

“For I really have the idea that the youth (…), that they more often have complaints of sadness. In some subgroups it’s ‘hot’ to have a psychologist, in others it’s the exact opposite. Some people come and say: “Everyone in my class has a psychologist and I haven’t. There must be something wrong with me. I need a psychiatrist too”. Then I start to wonder. Do young people really need to have complaints of sadness in order to fit in their group, to belong.” (A1)

Two other participants expressed a more opposite idea, and thought that young adults feel ashamed to be open about their complaints of sadness to family or friends and therefore consult a general practitioner. Instead of a skeptical attitude, the GPs are inclined to be extra watchful towards young adults.

“It happens to everybody once in a while, but if you fly off the handle at such a young age … Do you know what I think? Some people consult their GP for every single fart that’s bothering them. But many people don’t and that certainly includes most young people. They are reluctant to be open about it. Because it’s ‘not cool’ of course.” (A5)

Other characteristics of young adults the participants mentioned were flexibility and vulnerability. This can be negative because not much is needed to bring them in a negative mood, as well as positive because some small advice can already be helpful. Another participant said that young people have the best chance for recovery because they are still able to adjust their life and because they are suitable for therapy.

Two experienced GPs argued that young adults are a vulnerable group because they are entering a new life phase in which they have to be more independent. They assume this can be troubling because young people nowadays are raised in a more protective manner. They are not used to deal with setbacks.

“So yes, what strikes me is that a lot of young people have lived with their parents for a very long time and see their parents as some sort of friend, not as a parent anymore. Yes, maybe I am treading on thin ice right now …. But they have always been pampered. The only thing they’ve ever heard is appraisal: you’re great; well done; you’re so good; you’re the best. But … no one is perfect. It’s a good thing to fall flat on your face, to fail once in a while. Let me put it this way; it strikes me that many people, young people in particular, cannot cope well with setbacks and disappointments.” (GP4)

Suicidal thoughts, earlier treatment in psychiatry, and family members with depression made the participants more alert. This can lead a GP to a quick referral to psychiatry, giving the diagnosis of depression and starting with antidepressants sooner. Six participants, three alumni and three more experienced GPs, mentioned this. Familiarity and awareness of family members with depression only occurred in the GPs who had their own practice.

Finally, from the interviews, it appeared that the participants were also influenced when patients came up with their own diagnoses. One participant mentioned that especially students in social studies tend to diagnose themselves. The risk of this “self-diagnosing” is that GPs become biased, they said, especially with subjective complaints like complaints of sadness.

### The Drafted Typology

**FIGURE 2:**
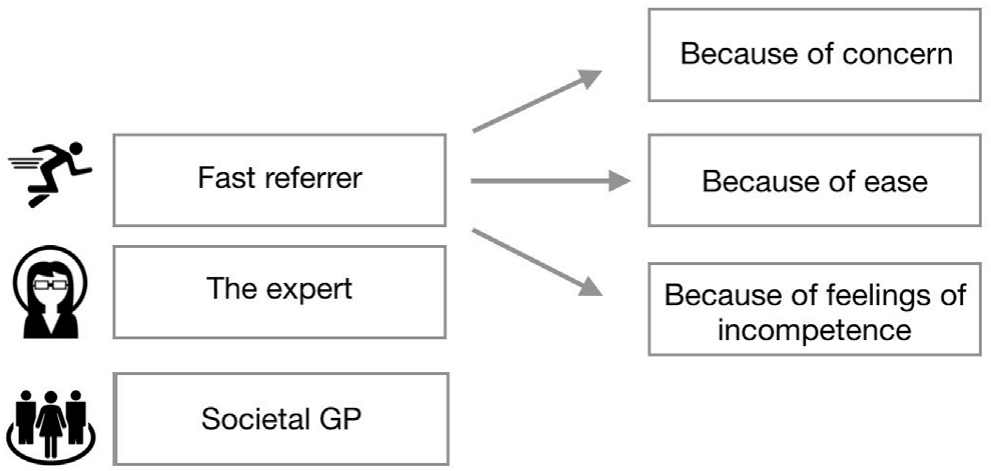
Typology of GPs.

Based on the results of this research—how the participants dealt with the subject—we created a typology of GPs (Figure 2). In this typology, we distinguish how the interviewed GPs were influenced by social factors, personal factors, professional factors, and patient characteristics. The first type is the fast referrer. It can be divided again into three subtypes regarding the reasons the GP has for referring the patient. The first one is *the referrer because of concern*. For example, there was a participant who experienced a suicide of someone she just referred to psychiatry some time before. For her, this experience is a reason to refer almost everybody with complaints of sadness to psychiatry. “Better overestimating than under estimating”, this GP argued. The second one is *the fast referrer because of ease*. A GP of this type, for example, was convinced that it is not the GP’s task to deal with complaints of sadness. GPs of this type were not very keen on the subject either. The third one is *the fast referrer because of feelings of incompetence*. These were mostly young doctors who said they lacked knowledge and training in how to deal with complaints of sadness. Therefore, it felt better for them to refer to another discipline.

The second type is *the expert*. This was the GP type that was interested in and felt competent with mental problems and therefore also tended to treat this kind of patient themselves. They used the DSM for this kind of problems and, if necessary, started antidepressants and sometimes even therapy by themselves.

The third type is *the societal GP*. This GP type considered that their responsibility may transcend pure “medical” problems and also see an important role for themselves on a more societal level: as someone with whom you can discuss life questions and existential difficulties. According to this type, in our secularized society, a GP also has the role of a “pastor” with whom people can discuss their problems. A GP of the “societal” type also argued that it is very important to abstain from sticking labels on people, especially on young adults.

## Discussion

### Macro Versus Micro Level

The results show that participating GPs strongly differ in the way they deal with complaints of sadness among young adults although they are all inclined to act proactively. An important finding was the diagnostic struggle the participants showed. There seems to be a paradox in the way GPs think about de-medicalization on a macro level and the way they are proactively acting on a micro level. On a macro level, the interviewed participants all recognized the social factors that may lead to an increase in complaints of sadness among young adults. They mentioned the importance of refraining to stick medical labels on patients too easily and the importance of alternative perspectives instead of just the medical perspective. On a micro level, however, with a patient actually sitting in front of them, all GPs want to do something, although all the participants acted differently and they clearly “struggled” with it. Standards such as the DSM and the derived NHG standard for depression seem to contribute to this. Dutch GPs are more or less bound to the DSM and the derived NHG standards, which include medical and pharmacotherapeutic guidelines. The guidelines are held in high regard by Dutch GPs (Schäfer et al., 2010). The criteria of the DSM (and the derived NHG standards) only involve the individual (complaints). They are not being placed in a broader psycho-social context. This is part of the medical paradigm that, according to psychologist Verhaeghe et al. (2013), makes it hard to have a more societal view on the difficulties people are experiencing. The medical paradigm with corresponding language (disorders) and the subsequent goal of “treatment” (discipline) lead to a redefining and expansion of what we see as “sick” (Devisch, 2013).

### Twaddle and Parsons

For a better understanding of the difference between a complaint of sadness and a recognized depression, a clear definition of the concepts disease, illness, and sickness can be useful. This full trial was firstly applied by Twaddle (1968). The distinction between disease, illness, and sickness has become commonplace in medical sociology, medical anthropology, and philosophy of medicine (Hofmann and Hofmann, 2002). According to Twaddle, disease is defined as a “health problem that consists of a physiological malfunction that results in an actual or potential reduction in physical capacities and/or a reduced life expectancy” (Twaddle, 1968, p. 8). Illness, on the other hand, is defined as “a subjectively interpreted undesirable state of health. It consists of subjective feeling states, perceptions of the adequacy of the bodily functioning, and/or feelings of competence” (Twaddle, 1968, p. 10). Sickness is defined as “a social identity”. It is the poor health or the health problem(s) of an individual defined by others with reference to the social activity of that individual” (Twaddle, 1968, p. 11). Receiving a diagnosis from a physician can legitimize the complaints. Something that was labeled as a complaint (illness) before is from then on a disease (Jutel, 2009). This has also been emphasized by Parsons (1951), who coined the classic concept of the “sick role” to define illness from a sociological perspective. He argued that being ill was not only a biological condition, but also a social role with a set of norms and values assigned to it. According to Parsons (1951), seeking for medical care is part of the sick role. A physician has the exclusive right to legitimize a sick role: an illness (“a subjectively interpreted undesirable state of health”) becomes transformed into a disease when this is considered applicable. Doctors can in this way be seen as “moral entrepreneurs” (Becker, 1963).

### The Semi-Legitimized Sick Role

**FIGURE 3:**
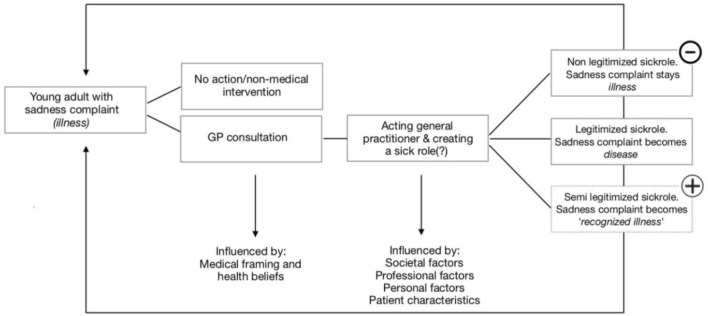
Conceptual model with the introduced semi-legitimized sick role.

Because the participants in the present study always undertook some kind of action based on the complaint of sadness, we introduce the terms “semi-legitimized sick role” and the “recognized illness.” The developed conceptual model in this research (Figure 3) displays this using the plus sign. This option applies for cases in which the GPs do act proactively and recognize the sadness complaints, but do not call it a depression yet. When they do label it as depression, a shift occurs between “illness” and “disease”, and the sick role (Parsons, 1951) is completely legitimized. The non-legitimized sick role where a complaint of sadness remains an illness after consulting the GPs is no longer relevant because the participants always acted in some way. This option (an illness that stays an illness) is therefore erased from the conceptual model, which is depicted in Figure 3 by the minus sign.

The results in this study are in line with the research of Mik-Meyer and Obling, 2012 who found that for a legitimate sick role, a traditional objective pathology in the body is not necessarily needed and subjective complaints (illness) may be enough for a GP to construct and legitimize the sick role. This happens by constructing and negotiating a sick role even when there is a lack of a clear-cut medical diagnosis and it is difficult to label a particular illness. This study also shows that “psychiatrization” is not an “exclusive problem” of only psychiatry and psychiatrists, but something that is also driven by non-psychiatric professionals, like GPs.

## Conclusion

### Findings

Based on our interviews with 13 GPs on how they handle complaints of sadness of youth, three typologies were identified: the fast referrer, the expert, and the societal GP. All participants endorsed de-medicalization on a macro level, but many had difficulties to put this into practice on a micro level.

According to the typology of GPs (Figure 2), the first subtype is *the fast referrer*: *the referrer because of concern*. This concern, coming from personal factors, could lead to refer most (young) people with complaints of sadness to psychiatry. This type therefore tends to legitimize the sick role relatively fast, with “illness” becoming “disease.” For the other two subtypes, the fast referrer because of ease and the fast referrer because of feelings of incompetence, it is not yet clear whether the sick role is going to be fully legitimized or semi-legitimized; this depends on the type and content of the follow-up contact.

In the second type, *the expert* tends to follow the more traditional script, working strictly according to the DSM and prescribing antidepressants themselves. This is why the sick role is legitimized relatively fast and easy whereby “illness” becomes “disease.”

The third type, *the societal* GP, prefers working without strictly adhering to the DSM and other standards. In this type, GPs seem to be most able to take off their medical glasses and show a more multifactorial view on complaints of sadness. Therefore, this type connects most to a course of de-medicalization that the Dutch Council for Health and Society (RvS, 2017) is pleading for. Also, this type does not tend to traditionally legitimize the sick role. Still, we did not want to do anything, that is why, in this study, we call this a semi-legitimized sick role where “illness” becomes “recognized illness.”

### Limitations

This research has an important restriction: the size of the included groups of GPs was too small to be able to say something about the influence of the change of the Dutch healthcare system in 2015.

### Practical Implications

Our research is useful in constructing how the sick role is (semi)legitimized for young adults with complaints of sadness by GPs and how GPs are influenced by different factors by using the typology of GPs (Figure 2). This also leads towards a better understanding in how GPs could be able and feel competent to take off their medical glasses and show a more multifactorial view on complaints of sadness. The obtained insights and knowledge from this study are a useful contribution to critical medical sociology but could also be used for (a more reflexive) practice in education and training of (future) GPs. For example, considering the finding that all GPs in our study are inclined to act and do something for youths presenting themselves with sadness complaints, future GPs could be reminded of the noble “art of doing nothing.” The principle of the (semi)legitimized sick role and the consequences of it may be helpful in learning to use time as both a diagnostic and a therapeutic tool (Heath, 2012).

### Future Research

Recommendations for further research would be to gain more information about the definition and prevalence of the different GP types. Another recommendation is to gain more information on the “recognized illness” with a semi-legitimized sick role that is introduced in this study, for example, by applying the constructs on recent research showing that adolescents who had received a mental health disorder diagnosis were often excluded from the labor market and education as young adults (Ringbom et al., 2021). By using the semi-legitimized sick role, contributions can be made to better understand how individuals are affected by diagnoses through the legitimizing of the sick role and how subsequently society is.

## Data Availability

The data supporting the conclusion of this article will be available on request to the corresponding author.

## Footnotes

1 The NHG clinical practice standards are most important in Dutch general practice. The NHG standard for depression is derived by the diagnostic criteria for depression in the Diagnostic and Statistical Manual of Mental Disorders (DSM IV).

2 The Dutch College of General Practitioners (Nederlands Huisartsen Genootschap, NHG) provides maximum scientific support for general practice, thus facilitating the work of the individual GP. One way in which the NHG provides support to GPs is through the development of medical and pharmacotherapeutical guidelines. The guidelines cannot be enforced legally, but are held in high regard by Dutch GPs. These guidelines contain recommendations about anamnesis, examination, treatment, prescription and referring (Scha˙fer et al., 2010).
